# New STLV-3 strains and a divergent SIVmus strain identified in non-human primate bushmeat in Gabon

**DOI:** 10.1186/1742-4690-9-28

**Published:** 2012-03-30

**Authors:** Florian Liégeois, Vanina Boué, Fatima Mouacha, Christelle Butel, Bertrand Mve Ondo, Xavier Pourrut, Eric Leroy, Martine Peeters, François Rouet

**Affiliations:** 1 UMI 233, «Trans VIH MI», Recherches Translationnelles sur le VIH et les Maladies Infectieuses Institut de Recherche pour le Développement (IRD), University of Montpellier 1, Montpellier, France; 2 Laboratoire de Rétrovirologie, Centre International de Recherches Médicales de Franceville, Franceville, Gabon; 3 UMR MIVEGEC, (IRD224-CNRS5290, University of Montpellier 1 & 2), Montpellier, France; 4 Centre International de Recherches Médicales de Franceville, BP 769, Franceville, Gabon, Africa

**Keywords:** SIV, STLV, Non-Human primate, Zoonotic infections, Bushmeat, Gabon

## Abstract

****Background**:**

Human retroviral infections such as Human Immunodeficiency Virus (HIV) or Human T-cell Lymphotropic Virus (HTLV) are the result of simian zoonotic transmissions through handling and butchering of Non-Human Primates (NHP) or by close contact with pet animals. Recent studies on retroviral infections in NHP bushmeat allowed for the identification of numerous Simian Immunodeficiency Viruses (SIV) and Simian T-cell Lymphotropic Viruses (STLV) to which humans are exposed. Nevertheless, today, data on simian retroviruses at the primate/hunter interface remain scarce. We conducted a pilot study on 63 blood and/or tissues samples derived from NHP bushmeat seized by the competent authorities in different locations across the country.

****Results**:**

SIV and STLV were detected by antibodies to HIV and HTLV antigens, and PCRs were performed on samples with an HIV or/and HTLV-like or indeterminate profile. Fourteen percent of the samples cross-reacted with HIV antigens and 44% with HTLV antigens. We reported STLV-1 infections in five of the seven species tested. STLV-3 infections, including a new STLV-3 subtype, STLV-1 and -3 co-infections, and triple SIV, STLV-1, STLV-3 infections were observed in red-capped mangabeys (*C.torquatus*). We confirmed SIV infections by PCR and sequence analyses in mandrills, red-capped mangabeys and showed that mustached monkeys in Gabon are infected with a new SIV strain basal to the SIVgsn/mus/mon lineage that did not fall into the previously described SIVmus lineages reported from the corresponding species in Cameroon. The same monkey (sub)species can thus be carrier of, at least, three distinct SIVs. Overall, the minimal prevalence observed for both STLV and SIV natural infections were 26.9% and 11.1% respectively.

****Conclusions**:**

Overall, these data, obtained from a restricted sampling, highlight the need for further studies on simian retroviruses in sub-Saharan Africa to better understand their evolutionary history and to document SIV strains to which humans are exposed. We also show that within one species, a high genetic diversity may exist for SIVs and STLVs and observe a high genetic diversity in the SIVgsn/mon/mus lineage, ancestor of HIV-1/SIVcpz/SIVgor.

## **Background**

Simian Immunodeficiency Viruses (SIV) and Simian T-cell Lymphotropic Viruses (STLV) infect a plethora of non-human primates (NHPs) in sub-Saharan Africa [1-16]. These viruses have crossed the species barrier on multiple occasions, probably by direct exposure by humans to infected blood and/or tissues when handling and butchering NHP bushmeat or from pet animals [17]. SIVcpz*Ptt* from chimpanzees and SIVgor from gorillas in west central Africa are the precursors of Human Immunodeficiency Virus type 1 (HIV-1) group M, N, O and P [18-20], and SIVsmm from west African sooty mangabeys (*Cercocebus atys*) are the ancestors of the different groups (A to H) of Human Immunodeficiency Virus type 2 (HIV-2) [21].

To date, five types of Primate T-cell Lymphotropic Virus (HTLV and STLV) have been identified (PTLV-1 to −5) [12], and three of them, PTLV-1 to −3, infect both simians and humans. No simian analogue has been found to the recently discovered HTLV-4 [12], while a human counterpart has yet been reported for STLV-5, which infects an Asian monkey *(Macaca arctoide)*. The different subtypes of HTLV-1 to −3, originated also from apes and/or monkeys [22-25]. STLV-1 has been documented in a wide variety of old world monkey species and apes from sub-Saharan Africa and Asia, but STLV-3 has only been identified in African NHPs [12,13,26,27]. The different STLV-1 and -3 subtypes represent geographic rather than species specific clusters demonstrating a high capacity of cross-species transmissions among NHP [12,13]. Moreover, STLV-1 viruses are interspersed within the different HTLV-1 subtypes (A to H), and the recently discovered HTLV-3 strains are also closely related to STLV-3 strains from NHPs in the same geographic areas, indicating also numerous cross-species transmissions between NHP and humans [25,28-31].

In contrast to STLV, each NHP species is generally infected with a species-specific SIV, i.e. multiple strains from the same host species form a monophyletic clade. This was used to establish the SIV nomenclature that names the various SIVs by adding a three letters code of their common name indicating the primate species of origin (e.g., SIVcpz from chimpanzee, SIVsmm from sooty mangabey). In some cases, closely related monkey species harbour also closely related SIVs, suggesting that some of these viruses may have coevolved with their hosts for an extended period of time or that SIVs could be transmitted preferentially according to an host-switching model, e.g., l’hoest and sun-tailed monkeys from the l’hoesti superspecies, the four species of African green monkeys (genus *Chlorocebus*), or SIVs from arboreal *Cercopithecus* species [32-34]. A single NHP species can also be infected by two different SIVs, e.g. SIVmnd-1 and −2 in mandrills which are separated by the Ogooue River, but co-circulating SIV variants have also been observed, e.g. SIVmus-1 and −2 in Cameroonian mustached monkeys *(C. cephus cephus)*[3,35]. There are also many examples of cross-species transmissions of SIVs between NHPs sharing the same habitat, e.g. SIVagm infecting African green monkeys has been transmitted to Patas monkeys in West Africa and to yellow and chacma baboons in Eastern and Southern Africa [36-38]. Cross-species transmission followed by recombination between different SIV strains can also occur, as demonstrated for SIVmus-2 infecting mustached monkeys from Cameroon, a virus resulting from the recombination of SIVgsn infecting greater spot nosed monkeys and SIVmus infecting mustached monkeys [3]. SIVcpz infecting chimpanzees is another example of cross-species transmission, followed by recombination between SIVrcm from red-capped mangabeys and SIVgsn from greater spot nosed monkeys [39]. These observations indicate that both cross-species transmission and co-infections, followed (or not) by recombination events with highly divergent lentiviral strains are possible. Overall, the evolutionary history of NHP lentiviruses has been driven by these successive events over an extended period of time.

During the last two decades, numerous studies have been conducted on wild living or captive NHPs as well as, although to a lesser extent on NHP bushmeat, broadening considerably our knowledge on simian retroviruses. Moreover, the recent identifications of the new HIV-1 group P closely related to SIVgor, the new HTLV-1 and HTLV-3 variants, and the simian foamy viruses in Cameroonian hunters with high nucleotide identity to STLV’s from monkeys hunted in this region, suggest relatively recent cross-species transmissions. These findings underline the fact that zoonotic retroviral infections are still ongoing in persons exposed to NHPs [20,25].

Nevertheless, our actual knowledge on the genetic diversity, their evolutionary history and the extent of human exposure to simian retroviruses remain incomplete mainly due to limited geographical and/or animal sampling. In this study, conducted in Gabon, we identified multiple retroviral infections, co-infections, and new SIV and STLV lineages in a small sample size of NHPs bushmeat, thereby confirming their wide diversity and complex evolutionary history.

## **Results**

### **STLV and SIV antibodies in non-human primates**

**Figure 1:**
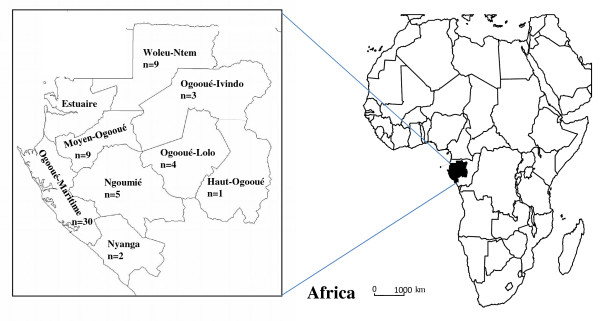
**Geographical distribution of NHP bushmeat samples in Gabon. Haut-Ogooué** (HO) n = 1 (*C. cephus*), **Moyen Ogooué** (MO) n = 9 (eight *C. cephus*, one *C. torquatus*), **Ngounié** (NG) n = 5 (three *C. cephus*, two *C. nictitans*), **Nyanga** (NY) n = 2 (one *C. cephus*, one *C. nictitans*), **Ogooué Ivindo** (OI) n = 3 (one *C. cephus*, two *C. nictitans*), **Ogooué Lolo** (OL) n = 4 (four *C. cephus*), **Ogooué Maritime** (OM) n = 30 (*one Lophocebus albigena*, six *C. cephus*, ten *C. nictitans*, six *C. pogonias*, seven *C. torquatus*), **Woleu-Ntem** (WN) n = 9 (four C*. cephus*, one *C. nictitans*, two *M. sphinx*, one *C. neglectus*, one *C. pogonias*).

Bushmeat samples from monkeys, belonging to seven species, were obtained in villages from different areas in eight out of the nine provinces in Gabon (Figure 1); the samples encompass 28 mustached monkeys (*C. cephus*), 16 greater spot nosed monkeys (*C. nictitans)*, 8 crested mona’s (*C. pogonias*), 7 red-capped mangabeys (*C. torquatus)*, 2 mandrills (*M. sphinx)*, 1 DeBrazza monkey (*C. neglectus)* and 1 grey-cheeked mangabey (*L. albigena*). Overall, 30/63 (47.6%) samples were collected in the Ogooue Maritime province. Seven samples were collected from juvenile monkey carcasses. A total of 21 whole blood samples (~44%) tested positive or indeterminate with the HTLV-1/HTLV-2 INNOLIA assay, as summarized in Table 1. Different INNOLIA profiles were obtained ranging from HTLV-1-positive (n = 7), dual HTLV-1/-2-positive (n = 1), HTLV positive but untypable (n = 4), to indeterminate (n = 9). Collectively, positive HTLV cross-reactive antibodies were detected in five out of seven NHP species studied. All seven samples from juvenile monkeys were negative.

**Table 1:** **Detection of HTLV-1 and HTLV-2 cross reactive antibodies and*tax*PCR results in non-human primates in Gabon**

**Species (common name)**	**No. of animals tested**	**Results of the InnoLIA-HTLV-1/2 confirmatory assay**						**No. of blood samples tested**	**No. of tissue samples tested**	**No. of positive *tax* PCRs for**			**No. of negative*****tax***** PCR**
		**HTLV-1**	**HTLV-2**	**HTLV 1 + 2**	**HTLV Unt**	**HTLV Ind**	**HTLV negative**			**STLV-1**	**STLV-3**	**STLV-1 + 3**	
*Lophocebus albigena* (Grey cheeked mangabeys)	1	0	0	0	0	1	0	1	0	0	0	0	1
*Cercopithecus cephus* (Mustached monkeys)	25	3	0	0	1	5	16	9	3	3	0	0	9^a^
*Cercopithecus neglectus* (De brazza’s monkeys)	1	0	0	0	0	0	1	0					
*Cercopithecus nictitans* (Greater spot nosed monkeys)	14	2	0	1	0	3	8	6	3	6	0	0	3^b^
*Cercopithecus pogonias* (Crested mona monkeys)	3	0	0	0	1	0	2	1	4	1	0	0	4^c^
*Mandrillus sphinx* (Mandrill)	2	2	0	0	0	0	0	2	0	2	0	0	0
*Cercocebus torquatus* (Red- capped mangabeys)	2	0	0	0	2	0	0	2	5	1	3	1	2^d^
Total	48	7	0	1	4	9	27	21	15	13	3	1	19

^a^Including the three tissue samples; ^b^Including the three indeterminate blood samples; ^c^Including three tissue samples; ^d^Including two tissue samples.

Four samples cross-reacted clearly with at least two HIV antigens in the INNOLIA HIV test (Table 2), i.e. one from *C. cephus*, one from *M. sphinx* and two from *Cercocebus torquatus*. Three additional samples showed either a cross-reactivity with only one HIV antigen (twice *C. cephus*) or a very weak cross-reactivity with two HIV antigens (one *C. nictitans*). Interestingly, only the four samples presenting an HIV-like profile tested positive with their specific V3 loop peptides ELISA assay (0.5 < Optical Density > 1.4), i.e. one *C. cephus*, one *M. sphinx* and two *C. torquatus* (Table 2)*.* Overall, positive HIV cross-reactive antibodies were detected in four of the seven NHP species and no juvenile monkeys were HIV sero-reactive.

**Table 2:** **Detection of HIV-1 and HIV-2 cross reactive antibodies and partial*pol*PCR results in non-human primates in Gabon**

**Species(Common name)**	**No. of animals tested**	**Results of the InnoLIA-HIV-1/2 confirmatory assay**			**Peptides results**	**V3 ELISA**	**No. of blood samples tested**	**No. of tissue samples tested**	**No. of samples positive for partial *pol* sequences**	**No. of samples negative for *pol* sequences**
		**HIV like^a^**	**HIV Ind^b^**	**HIV negative**	**pos**	**neg**				
*Lophocebus albigena* (Grey cheeked mangabeys)	1	0	0	1	0	1	0			
*Cercopithecus cephus* (Mustached monkeys)	25	1	2	22	1	24	3	3	1	5^c^
*Cercopithecus neglectus* (De brazza’s monkeys)	1	0	0	1	0	1	0			
*Cercopithecus nictitans* (Greater spot nosed monkeys)	14	0	1	13	0	14	1	3	0	4
*Cercopithecus pogonias* (Crested mona monkeys)	3	0	0	3	0	3	0	4	0	4
*Mandrillus sphinx* (Mandrills)	2	1	0	1	1	1	1	0	1	0
*Cercocebus torquatus* (Red-capped mangabeys)	2	2	0	0	2	0	2	5	5	2^d^
Total	48	4	3	41	4	44	7	15	7	15

^a^Plasma samples were scored as HIV-like when they reacted with at least two HIV antigens and had a band intensity equal to or greater than the assay cutoff (+/−) lane; ^b^Samples that reacted less strongly but still visibly with one or more HIV antigens were classified as indeterminate (Ind); ^c^Including the three tissue samples; ^d^Including two tissue samples.

### **Molecular characterization and phylogenetic analyses of STLV**

#### ***Confirmation of STLV infection by confirmatory and discriminatory PCR analyses of the tax gene***

Among the 21 samples positive or indeterminate for HTLV antibodies, all seven HTLV-1-positive samples as well as the sample with an HTLV-1/-2 profile were amplified with the STLV-1 specific *tax* primers. Among the four untypable samples, one was amplified with the STLV-3 specific *tax* primers; one was reactive with both STLV-1 and STLV-3 specific *tax* primers; and two could not be amplified. All nine indeterminate samples were negative by generic and type specific PCRs. Among the 15 tissue samples, five were amplified with STLV-1 specific *tax* primers, two with STLV-3 specific *tax* primers; and eight were negative by generic and type specific PCRs. The minimal prevalence of STLV infection is thus 17/63 (26.9%). All *tax* PCR results are summarized in Table 1.

**Figure 2:**
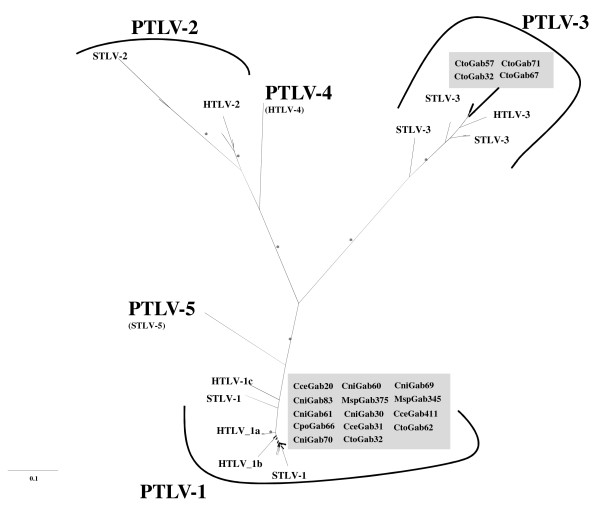
**Phylogenetic analysis of STLV*tax-rex*sequences.** PTLV phylogeny inferred using 220-bp *tax-rex* sequences. Reference sequences used were as follow: **HTLV-1** (RKI3Ger_AF042071, TSP-1_M86840, HS35_AF033817, BOI_L36905, MT2_L03561, ATK-1_j02029, ATL-YS_U19949, WHP_AF259264, EL_M67514, MEL5_L02534), **STLV-1 (**Cce01CM1374_ AM746629, Cce01CM3020_ AM746632, Cpo01CM2324_ AM746633, Cpo01CM2230_ AM746634, Cni01CM4078_ AM746635, Cce01CM2141_ AM746636, Cce01CM1445_ AM746638, Cce01CM2205_ AM746639, Cni01CM2198_ AM746640, Mta 00cm265_AY496616, Tan90_AF074966, Cta01CMS75_AY496618, Cpo99cm190_AY496615, Cpo01cm1228_AY496612, Mnd 98cmB111_AY496617, Cni01cm1040_AY496608, Cag01cm1312_AY496614, Cag 01cm1135_AY496610, TE4_Z46900), HTLV-II (GAB_Y13051, GU_X89270, NRA-P_L20734, G12_l11456, Efe2_Y14365, G2_AF074965, RP329_AF326583, SP-WV_AF139382, k96_AF326584, Mo_M10060), **STLV-2** (PanP_U90557, PP1664_NC001815), **HTLV-3** (Pyl 43_DQ020492), **STLV-3** (Lal01CM2008_ AM746647, Lal01CM4009_ AM746650, Cag02CM4101, PPA-F3_AF5177775, PH969_Y07616, TGE-2117_AY217650, hyb2210_AF378162, CTO-604_NC003323, Pha7550_AF378160, Cag01cm1184_AY496593, Cni227_AF412120,Cni217_ AY039033), **HTLV-4** (1863LE_NC_011800), **STLV-5 (**MarB43_AY590142). Numbers correspond to internal branch support derived from 1000 bootstrap replicates (only values above ≥ 80% are shown and represented by an asterisk). Scale bar represents the number of nucleotide substitutions per site. Groves’ primate taxonomy nomenclature is used [40]. Non-human primates are coded using the first letter of the genus followed by the first two letters of the species name: Cni = *Cercopithecus nictitans*, Cce *= Cercopithecus cephus*, Msp *= Mandrillus sphinx*, Cto *= Cercocebus torquatus*, Cpo = *Cercopithecus pogonias*. Sequences in bold text and in grey rectangles are from the current study.

The phylogenetic relationships of the 220-bp *tax/rex* sequences from the STLVs obtained in this study are shown in Figure 2. All 14 STLV-1 strains fell in the cluster of PTLV-1 strains whereas the four STLV-3 strains clustered with PTLV-3 sequences. Interestingly, STLV-3 strains have only been identified in red-capped mangabeys (*C. torquatus*) with one strain, which seemed to be more divergent than other new STLV-3 strains.

The phylogenetic relationships of these new viruses within either STLV-1 or STLV-3 lineages were further analyzed based on LTR (STLV-1) and *tax*-pX-LTR (STLV-3) sequence comparison as described below.

#### ***Analysis of STLV-1 LTR sequences***

A 450-bp fragment of the LTR region was sequenced in 10 out of the 14 STLV-1-infected animals: one *M. sphinx*, four *C. cephus*, two *C. torquatus* and three *C. nictitans*. We failed to amplify the LTR region for four animals (one *M. sphinx*, one *C. pogonias* and two *C. nictitans*).

**Figure 3:**
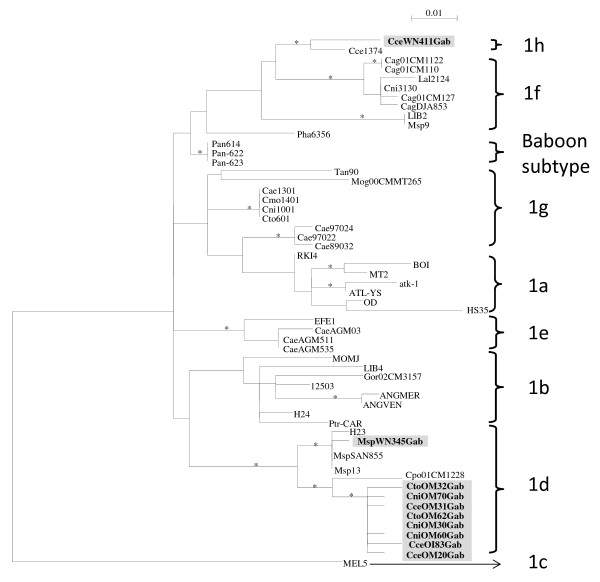
**Phylogenetic analysis of STLV-1 LTR sequences.** Inference of PTLV-1 phylogeny using 450-bp LTR sequences. Reference sequences used were as follow: **PTLV-1a** (BOI_L36905, MT2_Z31660, atk-1_J02029, ATL-YS_U19949, OD_U12805, OD_U12805, RKI4_AF054627), **PTLV-1b (**MOMJ_Z31659, LIB4_Y17019, Gor02CM3157_AY496635, 12503_L76309, ANGMER_AY026858, ANGVEN_AY026857, H24_L76308, PTR-CAR_AF384871), **PTLV-1c (**MEL5_L02534), **PTLV-1d (**H23_L76312, MSPSAN855_AF384870, MSP13_AF045933, Cpo01CM1228_AY496628), **PTLV-1e (**EFE1_Y17014, CAEAGM03_AY026848, CAEAGM511_AY026847, CAEAGM535_AY026846), **PTLV-1f (**Cag01CM1122__AY496630, Cag01CM1106_AY496629, Cag01CM1272_AY496633, CAGDJA853_AF384872, LIB2_Y17017, Msp9_AF045932,Lal01CM2124_AM712678, Cni01CM3130_AM712679, **PTLV-1g** (Tan90_AF074966, Mog00CMMT265_AY496636, CAE1301_AY026852, CMO1401_AY026853, Cni1001_AY026854, Cto601_AY026855, Cae97024_AY026849, Cae97022_AY026850, Cae89032_AY026851), **PTLV-1h** (Cce01CM1374_AM712677), **Baboon subtype** (Pha6356_Y13347, Pan614_AY026844, Pan622_AY026843, Pan623_AY026842). Numbers correspond to internal branch support derived from 1000 bootstrap replicates (only values ≥ 80% are shown and represented by an asterisk). Scale bar represents the number of nucleotide substitution per site. Groves’ primate taxonomy nomenclature is used [40]. Non-human primates are coded using the first letter of the genus followed by the first two letters of the species name: Cni *= Cercopithecus nictitans*, Cce = *Cercopithecus cephus*, Msp *= Mandrillus sphinx*, Cto *= Cercocebus torquatus*, Cae = *Chlorocebus aethiops,* Gor *= Gorilla gorilla,* Pha *= Papio hamadryas*, Pan *= Papio Anubis*, Mog = *Miopithecus ogoouensis*, Ptr *= Pan troglodytes*. Sequences in bold text and in grey rectangles are from the current study.

As for the phylogeny of the *tax-rex* region, nine out of the ten new STLV-1 LTR sequences clustered with the African PTLV-1 strains and more precisely with HTLV-1 subtype D (Figure 3). Among the HTLV-1 subtype D clade, the new STLV-1 strains from *C. torquatusC. cephus* and *C. nictitans* were closely related to each other and to previously described STLV-1 sequences from *Cercopithecus* monkeys from Cameroon [12,41,42]. The new STLV-1 strain from *M. sphinx* is close to the reported STLV-1 strains from mandrills in Cameroon and Gabon [12,41-43]. Interestingly, one STLV-1 strain from *C. cephus* (CceWN411GAB) was clustered with the *C. cephus* (Ccep1374) STLV-1 strain from a new subtype H characterized in Cameroon [12].

#### ***Phylogenetic analysis of STLV-3 tax-pX-LTR sequences***

**Figure 4:**
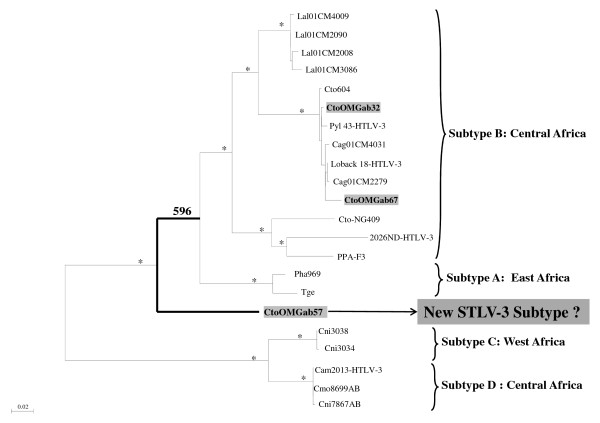
**Phylogenetic analysis of STLV-3*pX*-LTR sequences.** Inference of PTLV-3 phylogeny using 900-bp tax-pX-LTR sequences. Reference sequences used were as follow: **STLV-3** (Cto604_AF391797, Cto-NG409_AY222339, Ppa-F3_AF517775, TGE-2117_AY217650, Pha969_Y07616, Lal01CM2090_AM712666, Lal01CM3086_AM712667, Lal01CM4009_AM712672, Lal01CM2008_AM712673, Cag01CM4031_AM712670, Cag01CM2279_AM712669, Cmo8699AB_EU231644, Cni7867AB_EU152281, Cni3034_FJ957877, Cni3038_FJ957878), **HTLV-3 (**Pyl 43_DQ462191, Loback 18_EU649782, 2026ND_DQ093792, Cam2013_GQ463602). Numbers correspond to internal branch support derived from 1000 bootstrap replications (only values ≥ 80% are shown and represented by an asterisk except for the non-significant bootstrap percentage (59.6%) for the putative new STLV-3 subtype CtoOMGAB57). Scale bar represents the number of nucleotide substitution per site. Groves’ primate taxonomy nomenclature is used [40]. Non-human primates are coded using the first letter of the genus followed by the first two letters of the species name: Cto = *Cercocebus torquatus*, Cag = *Cercocebus agilis*, Lal = *Lophocebus albigena,* Cni = *Cercopithecus. Nictitans,* Ppa *= Papio hamadryas papio,* Tge = *Therppithecus gelada,* Pha = *Papio hamadryas*. Sequences in bold text and in grey rectangles are from the current study.

The 900-bp fragment spanning the *tax-rex* and pX-LTR region was sequenced from three of the four STLV-3 infected red-capped mangabeys (Figure 4). We failed to amplify STLV-3 *tax*-pX-LTR sequence from one *C. torquatus* sample. Two new STLV-3 sequences (CtoOMGab32, CtoOMGab67) clustered within the PTLV-3 subtype B and formed a specific sub-clade together with the STLV-3 strains from wild caught Cameroonian *C. torquatus* and *C. agilis* monkeys and with the HTLV-3 Pyl 43 and Loback 18 strains from humans (Figure 4) [12,41,44-46]. Interestingly, one new STLV-3 strain (CtoOMGab57) did not cluster with the previously identified PTLV-3 strains and did not group at a significant bootstrap level within any of the actual described STLV-3 subtypes, strongly suggesting the identification of a new lineage (Figure 4).

### **Molecular characterization and phylogenetic analyses of SIV**

#### ***Confirmation of SIV infection by PCR analyses of the partial pol gene***

SIV infections were confirmed in four of the seven SIV seroreactive samples and in three of the 15 monkey tissue samples, including five *C. torquatus*, one *C. cephus* and one *M. sphinx*. Only the four samples presenting an HIV-like profile and tested positive with their specific V3 loop peptides ELISA were SIV PCR positive, which is consistent with a better specificity and sensitivity of the in-house EIA specific test compared to SIV antibodies/HIV antigens cross reactivity tests as previously reported [2].

**Figure 5:**
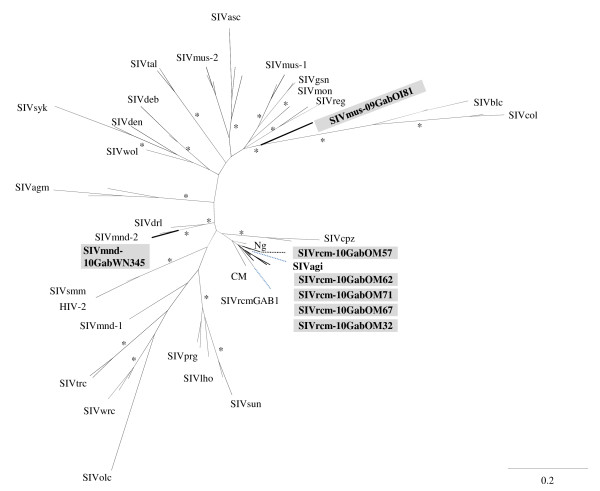
**Phylogenetic analysis of partial SIV*pol*sequences.** Phylogenetic relationships of the newly derived SIVs in partial *pol* sequences to representatives of the other SIV lineages. Reference sequences used were as follow: SIVwrc*Pbt* (western red colobus monkey*, Piliocolobus badius temminckii)* 05GMX02_AM937062, SIVwrc*Pbb (*western red colobus monkey*, Piliocolobus badius badius)* 98CI04_AM713177, SIVwrc*Pbb*97CI14_AM745105, SIVtal (talapoin monkey, *Miopithecus ogoouensis)* 00CM266_AY655744, SIVtal01CM8023_AM182197, SIVmon (mona monkey, *Cercopithecus mona)* 99CMCML1_AY340701, SIVgsn (greater spot nosed monkey, *Cercopithecus nictitans*) 99CM166_AF468659, SIVgsn99CM71_AF468658, SIVsyk (Sykes monkey, *Cercopithecus albogularis*) KE51_AY523867, SIVsyk173_L06042, SIVmus-1 (Mustached monkey, *Cercopithecus cephus*) 01CM1085_AY340700, SIVmus- 1.01CMCM1239_EF070330, SIVmus-2 01CM1246_EF070329, SIVdeb (DeBrazza monkey, *Cercopithecus neglectus*) 99CMCM40_AY523865, SIVdeb99CMCM5_AY523866, SIVden (Dents monkey, *Cercopithecus denti*) CD1_AJ580407, SIVsun (Suntailed monkey, *Cercopithecus solatus*) L14_AF131870, SIVlho (L’Hoest monkey, *Cercopithecus l’hoesti*) 7_AF075269, SIVmnd-1 (Mandrill, *Mandrillus sphinx*) Gab1_M27470, SIVmnd-2 M14_AF328295, SIVdrl (Drill, *Mandrilus leucophaeus*) FAO_AY159321, SIVolc (olive colobus monkey, *Procolobus verus*) 97CI12_FM165200, SIVcol (black and white colobus monkey, *Colobus guereza*) CGU1_AF301156, SIVagmver (Afican green monkey, *Chlorocebus vervet*)155_M29975, SIVagmgri (Afican green monkey, *Chlorocebus grivet*) 677_M66437, SIVagmTAN (Afican green monkey, *Chlorocebus tantalus*) 1_U58991, SIVrcm (red-capped mangabey, *Cercocebus torquatus*) GAB1_AF382829, SIVrcmNG411_AF349680, SIVrcm-02CM8081_ HM803689, SIVagi (Agile mangabey, *Cercocebus agilis*) 00CM312_ HM803690, SIVcpz*Pts* (Chimpanzee, *Pan troglodytes schweinfurtii*) TAN1_AF447763, SIVcpz*Ptt*_(Chimpanzee, *Pan troglodytes troglodytes*) AF103818, SIVsmm (Sooty mangabey, *Cercocebus atys*) US-H9_M80194, HIV-2Rod_ M15390, SIVreg (Red eared guenon, *Cercopithecus erythrotis*) REG016_ HM363408, SIVreg-REG001_ HM363406, SIVblc (Black colobus monkey, *Colobus satanas*) BCM91_ HM363421, SIVbcm-BCM307_ HM363423, SIVprg *(*preussis monkey*, Cercopithecus preussi insularis*) PRG138_ HM363425, SIVprg-PRG056_ HM363424, SIVwol (Wolf’s monkeys, *Cercopithecus mona wolfi)* 09DRC5_JN020273, SIVtrc (Tshuapa red colobus (*Piliocolobus tholloni)* 09DRC67_JN020274, SIVtrc-09DRC73_JN020275, SIVasc (redtailed monkey, *Cercopithecus ascanius)* 09DRC93_JN020276, SIVasc-09DRC99_JN020277, SIVasc-10DRC110_JN020278, SIVasc-10DRC149_JN020279. The newly identified SIV strains in this study are highlighted in grey rectangles and in bold text.. Within the SIVrcm clade, acronyms CM, Ng and Gab corresponded to the geographical origin of these SIVrcm strains (CM = Cameroon, Ng = Nigeria and Gab = Gabon). The unrooted trees were inferred from 300 bp nucleotides. The analyses were performed using discrete gamma distribution and GTR model. The starting tree was obtained by using phyML. One thousand bootstrap replications were performed to assess confidence in topology (only values ≥ 80% are shown and represented by an asterisk). Scale bar represents the number of nucleotide substitution per site.

The phylogenetic tree analysis revealed the presence of a new SIV variant in mustached monkey (*C. cephus*) (Figure 5) whereas the new SIV sequences from *M. sphinx* and *C. torquatus* fell within their respective lineages. All new SIVrcm strains from Gabon formed a specific sub-clade with the previously described SIVrcmGab1 from a captive red-capped mangabey from Gabon and interestingly with SIVagi from *Cercocebus agilis* identified in Cameroon. However, the phylogenetic topology was not supported by a significant bootstrap value probably caused by the small fragment size analyzed.

#### ***Phylogenetic analyses of partial SIVrcm gag, pol and env sequences***

**Figure 6:**
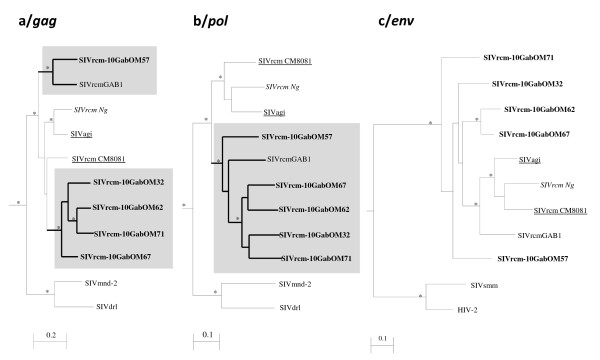
**Phylogenetic analysis of partial SIVrcm*gag, pol*and*env*sequences.** Phylogenetic relationships of the newly derived SIVrcm sequences in partial a/gag, b/*pol* and c*/env* sequences to the previously described SIVrcm strains from Cameroon, Nigeria and Gabon as well as with SIVagi from Cameroon. Reference sequences used were as follow: SIVmnd-2 M14_AF328295, SIVdrlFAO_AY159321, SIVrcmGAB1_AF382829, SIVrcmNG411_AF349680, SIVrcm-02CM8081_ HM803689, SIVagi-00CM312_ HM803690, SIVsmmUS-H9_M80194, HIV-2Rod_ M15390. The newly identified strains in this study are in bold text and specific sub-clades are shown in grey rectangles for *gag* and *pol*. The rooted trees were inferred from 470, 1800, 500 bp nucleotides for *gag*, *pol* and *env* respectively with representatives of the other SIV lineages. Only sequences of interest are showed with the closer SIVs outgroup in the complete tree topology (SIVmnd-2 and drl for *gag* and *pol* trees, SIVsmm and HIV-2 for *env* tree). SIV strains from Cameroon and Nigeria are underlined and in italic respectively. The analyses were performed using discrete gamma distribution and GTR model. The starting tree was obtained by using phyML. One thousand bootstrap replications were performed to assess confidence in topology (only values ≥ 80% are shown and represented by an asterisk). Scale bar represents the number of nucleotide substitution per site.

As depicted in Figure 6, for the 470 bp *gag* fragment, SIVrcm-10GabOM32, 62, 67 and 71 formed a sub-clade and are closest to SIVrcm from Cameroon and Nigeria and SIVagi, whereas SIVrcm-10GabOM57 was more closely related to SIVrcmGab1 from Gabon. In the 1800 bp *pol* fragment, all SIVrcm strains from Gabon clustered together (although SIVrcm-10GabOM57 and SIVrcmGab1 occupied a basal position in the tree topology) whereas SIVrcm/agi strains from Cameroon and Nigeria formed a separate sub-clade. In the 500 bp *env* fragment, all SIVrcm strains from Gabon, Cameroon and Nigeria as well as SIVagi clustered together but no sub-clade appeared clearly. More detailed analysis of the sequenced *pol* fragment did not reveal inter SIVrcm recombinants or with other SIV lineages.

#### ***Phylogenetic analyses of partial SIVmus env sequences***

**Figure 7:**
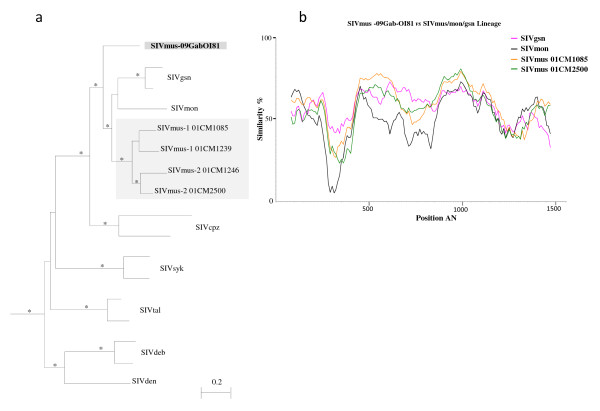
**Phylogenetic analysis of partial SIVmus-09OlGab81*env*sequences. a)** Phylogenetic relationships of the newly derived SIVmus-09GabOI81 sequences in partial *env* sequence to representatives of the other SIV lineages. Only sequences of interest are showed. Reference sequences used were as follow: SIVtal00CM266_AY655744, SIVtal01CM8023_AM182197, SIVmon99CMCML1_AY340701, SIVgsn99CM166_AF468659, SIVgsn99CM71_AF468658, SIVsykKE51_AY523867, SIVsyk173_L06042, SIVmus-1 01CM1085_AY340700, SIVmus-1.01CMCM1239_EF070330, SIVmus-2 01CM2500_EF070331, SIVmus-02 01CM1246_EF070329, SIVdeb99CMCM40_AY523865, SIVdeb99CMCM5_AY523866, SIVdenCD1_AJ580407, SIVcpz*Pts*_AF447763, SIVcpz*Ptt*_AF103818. The newly identified SIVmus strain in this study is highlighted in grey rectangle and in bold text, and reference strains are in black except for SIVmus from Cameroon that is in a grey rectangle. The rooted trees were inferred from 1900 nucleotides. The analyses were performed using discrete gamma distribution and GTR model. The starting tree was obtained by using phyML. One thousand bootstrap replications were performed to assess confidence in topology (only values ≥ 80% are shown and represented by an asterisk). Scale bar represents the number of nucleotide substitution per site. **b)** Similarity plots of SIVmus partial *env* nucleic acids sequence showing similarities between SIVmus-09GabOI81 *versus* other SIVs representative for the SIVmus/mon/gsn lineages, obtained with a sliding window of 160 nucleic acids (na) moved in steps of 10 na . The vertical axis shows the percentage of similarities and the horizontal axis shows the nucleic acid positions.

To further document the existence of a putative new SIVmus variant in mustached monkeys from Gabon, we compared a 1900 bp SIVmus-09GabOI81 partial *env* sequence with *env* sequences from all other SIV lineages available in GenBank. SIVmus-09GabOI81 partial *env* sequence was significantly closer to SIVmon/gsn/mus lineage than to the other SIV lineages (Figure 7a). All SIV strains composing the SIVmon/gsn/mus lineages, including the new SIVmus-09GabOI81 were equidistant to each other with nucleotide sequence identities varying from 68 to 73% (data not shown). These data were confirmed by the Simplot analysis (and by bootscan analysis as well, data not shown), which also showed that the new strain was not a recombinant virus with any of the other know SIVgsn/mus/mon strains or another SIV lineage (Figure 7b). Although SIVmus-09GabOI81 fell within the SIVmon/gsn/mus lineage from Cameroonian arboreal *Cercopithecus* monkeys, in both *pol* and *env* tree topologies, this lentivirus occupied a basal position within this lentiviral lineage. Overall, these results suggested that mustached monkeys in Gabon are infected with a SIVmus lineage that is different from SIVmus-1 and 2 identified in Cameroonian monkeys [3].

### **SIV and STLV co-infections**

In this study, co-infections with SIV and STLV were only observed in red-capped mangabeys, with four (on a total of seven) animals dually infected with SIV and STLV-1 or −3 (CtoOMGab57, 62, 67 and 71) and one triply infected with SIV, STLV-1 and STLV-3 (CtoOMGab32).

## **Discussion**

Simian retroviruses may easily jump the species barrier leading to human retroviral infections such as HIV/AIDS, thereby causing considerable damages for human health. Numerous studies have documented retroviruses in NHP species but most of them have been conducted in captive, breeding colonies, and to a lesser extent on wild living monkeys. Although these reports contribute to our knowledge on genetic diversity of retroviruses and evolution in their natural hosts, they do not provide data on their prevalence and the extent of human exposure to these viruses [4,6,10,11,15,16,27,32,35-37,42,43,47]. Indeed, the most probable ways of zoonotic retroviral infections are related to hunting and butchering of NHPs or by injuries like bites or scratches caused by pet primates. Studies on retroviruses in NHP bushmeat are limited and have been only conducted in few sampling sites in Sierra Leone, Cameroon, Bioko Island, and the Democratic Republic of Congo (DRC) [2,5,12-14,25,41,48-51]. These studies showed heterogeneous exposure according to geographic areas, both in terms of the prevalence and the genetic diversity of circulating strains.

Here, we report a pilot study on NHP bushmeat in Gabon. At least 19 NHP species inhabit Gabon, including *CercopithecinaeColobinae* and apes [52]. Despite our small sample size (63 samples from seven different species), our work revealed the presence of new STLV-1 (n = 10) and STLV-3 (n = 3) strains, with STLV-1 infections in *C. cephus, C. nictitansMandrillus sphinx* and *C. torquatus*, and STLV-1 + −3 dual infections in *C. torquatus*. In addition, one *C. torquatus* was SIV/STLV-1/STLV-3 co-infected (triple retroviral infection). Finally, we were able to partially characterize a new SIV strain in one *C. cephus*.

Nine of the ten Gabonese STLV-1 strains fell consistently in the west Central Africa STLV-1 subtype D lineage. One strain (Cce WNGab411) from *C. cephus* clustered within the putative STLV-1 subtype H clade which was recently described in south Cameroon from the same species (Cce 01CM1374) [12]. The close relationship between these two STLV-1 strains may be explained by the small geographical distance separating them, which does not exceed 150 km. It also suggests that STLV-1 subtype H strains may be widely circulated among *C. cephus* and possibly also in other NHP species in Central Africa.

In our survey, STLV-3 strains were only detected in *C. torquatus* sampled in the Ogooue Maritime province, which is the geographic range where this species can be encountered, i.e. coastal areas from southeast Nigeria to Gabon*.* Two of them clustered strongly together with STLV-3 strains from *C. torquatus* and *C.agilis* identified in Cameroon and with the human Pyl 43 and Loback 18 HTLV-3 strains also isolated in Cameroon [12,41,44-46]. To date, HTLV-3 infections have only been reported in Cameroon. The identification of STLV-3 infected hunted NHPs in our study suggests that HTLV-3 strains could also be present in Gabon. In addition, we found a new STLV-3 strain that did not cluster within PTLV-3 subtypes known so far. This strain could represent a new STLV-3 subtype. Overall, our results indicate that at least two different STLV-3 lineages or subtypes (Cto OMGab67 and 32 *vs* Cto OMGab57) co-circulate within the same monkey species from the same geographical area.

All SIVrcm from Gabonese *C. torquatus* fell in the SIVrcm lineage and potential sub-clades in *gag* and *pol* genes were observed. Although we did not observe recombination events in the *pol* gene, we could not exclude that SIVrcm recombinant forms circulate within close red-capped mangabey groups such as reported within western red colobus in the Ivory Coast’s Taï forest [53]. Nonetheless, further investigations and analyses within different wild living red-capped mangabey groups will allow the epidemiological dynamic of this SIV to be determined. Besides, red-capped mangabeys are likely infected with a monophyletic SIV lineage independently of the natural barriers separating their habitat along the coast of the Guinean gulf. These data suggest a long-standing lentiviral infection among these animals. Interestingly, five out of the seven samples tested (~70%) from red-capped mangabeys were co-infected with both STLV and SIV raising the number of monkey species known to harbor retroviral co-infections, such as previously documented in a few other monkey species [5,7,9,13]. Of note, retroviral co-infections are probably underestimated since surveys characterizing the different existing simian retrovirus (SIV, STLV and Simian Foamy Viruses) from a same population are very scarce.

Finally, we described a new SIV strain infecting the *C. cephus cephus* subspecies (mustached monkeys) sampled north of the Ogooue River in Gabon. The *cephus* superspecies is composed of six species and 14 subspecies. In West Central Africa, one species (*C. cephus*) consisting of four subspecies are present: *C. c. erythrotys* in Cameroon above the Sanaga River and in Bioko Island*, C. c. cephode* south of the Ogooue River in Gabon*, C. c. ngottoensis* between the Sangha and the Congo-Oubangui Rivers in Congo and *C. c. cephus* inhabiting the largest part of west Central Africa from the south of the Sanaga River to the north of the Ogooue River and following the right bank of the Congo River [54].

As reported by Aghokeng *et al.* in Cameroon, two distinct lentiviruses, SIVmus-1 and SIVmus-2, co-circulate in the *C. cephus cephus* subspecies among animals living in the same geographic area [3]. SIVmus-1 and SIVmus-2 are each other’s closest relatives and form with SIVgsn from greater spot nosed monkeys (*C. nictitans*) and SIVmon from mona monkeys (*C. mona*) the SIVmus/mon/gsn lineage. Importantly, SIVmus-2 is a recombinant lineage and consists of alternating sequences of SIVmus, SIVgsn and an unknown SIV in Gag and Pol proteomes. In the present study, the SIVmus-09GabOI81 strain is at the root of the SIVmus/mon/gsn lineage, strongly suggesting a new SIV lineage into this major lineage. Mustached monkeys appear to be the first NHP carrying three distinct SIVs. The basal position of SIVmus strain from Gabon among this lineage suggests a possible ancestrality of this virus in this clade or a more ancient infection within mustached monkeys from Gabon. However, it cannot be excluded that more detailed genetic analysis of the host will reveal a different subspecies. Similarity plot and bootscan analyses showed that this new variant is not recombinant in the studied fragment. Nonetheless, to date, recombination events have not been observed within the SIVs *env* gene and only full-length genome analysis of this new SIVmus will clarify the genetic relationship with the other strains of the SIVmus/mon/gsn lineage, including the presence or not of a *vpu* gene. In addition, documenting and characterizing SIV infections within other *C. cephus* subspecies, but also within the same subspecies in other geographic areas, are required to further understand the genetic diversity of SIVs in mustached monkeys and their evolutionary history.

## **Conclusion**

Our study identified high prevalence of retroviral infections and new STLV and SIV strains in a small sample size of NHP bushmeat in Gabon. Our data emphasize the need to perform larger studies on NHP retroviral infections, with a larger number of samples, a wider diversity of species and subspecies in African countries where NHPs are hunted for bushmeat. Similarly, as in other countries in Central Africa, bushmeat provides also an important source of protein in household diets in Gabon. Although NHPs are less hunted than other mammals, they represent, even so, about 12% of hunters’ preys [55]. Different studies showed that recent transmissions of simian retroviruses to human still occur [20,25,47]. Thus, it is important to identify and characterize retroviruses that circulate in African NHP species in order to estimate which retroviruses can potentially cross the species barrier and infect the human population. Previous studies on simian retroviruses in NHP bushmeat showed heterogeneous exposure in terms of prevalence and genetic diversity, depending on geographic areas studied and SIV’s prevalence in predominantly hunted species, because SIV’s prevalence can vary per species. Here we show that also among the same species, a high genetic diversity exist for SIVs and STLVs according to geographic origins.

## **Methods**

### **Study sites and animals**

Whole blood (n = 48) and lymph node tissues (n = 15) were obtained from 63 wild-caught monkey carcasses seized by agents of the provincial direction of the Gabon’s Ministry of Water and Forests. Seven out of the 63 samples were collected from juvenile animals (<2 years). Sampling was performed in eight provinces of Gabon from September 2009 to June 2010 (Figure 1). Species were initially determined by visual inspection according to the Kingdon Field Guide to African Mammals [56] and the taxonomy described by Colin Groves [40]. All NHP samples were obtained with the authorization of provincial inspections of Water and Forests. The Gabonese Ministry of National Education, Higher Education and Scientific Research and Innovation approved this study with the authorization n° AR0031/09/MENESRESI/CENAREST/CG/CST/CSAR.

### **Serological testing**

All whole blood samples were tested for the presence of STLV and/or SIV antibodies with commercially available confirmatory tests, INNOLIA HTLV-1/2 and INNOLIA HIV 1/2 (Innogenetics, Ghent, Belgium). These line immunoassays are able to discriminate between HTLV-1 and HTLV-2 and HIV-1 and HIV-2 cross-reactive antibodies, as previously described [12,14]. In addition, for the detection of SIV antibodies, an in-house strain-specific ELISA based assay using SIV lineage-specific V3-loop as antibody capture antigens was performed [57]. The SIV V3-loop peptides were chosen according to the NHP species encountered in Gabon: SIVmnd, SIVgsn/mus/mon, SIVrcm, SIVdeb, SIVlhoest/sun, SIVcol, SIVcpzGab1 and Gab2.

### **DNA extraction, PCR and sequencing**

#### ***DNA extraction***

DNA was extracted from whole blood and/or tissues using Qiagen DNA extraction blood and tissues kits (Qiagen, Courtaboeuf, France). DNA integrity and monkey species were confirmed by amplification of glucose-6-phosphate dehydrogenase gene (G6PDH) and mitochondrial 12sRNA gene respectively as previously described (Additional file 1: Figure 1) [14,58]. In addition, for the animal OI81, 12sRNA PCRs were done separately using extracted DNA from both whole blood and lymph node to confirm the monkey species.

#### ***Molecular confirmation of STLV infection***

To confirm the presence of STLV infection in samples with HTLV cross-reactive antibodies, a diagnostic *tax-rex* PCR (220 bp) allowing generic as well as type-specific detection of PTLVs was done. The generic PCR shows high sensitivity in detecting all PTLV groups, whereas the discriminatory PCRs have high specificities to discriminate between PTLV-1, PTLV-2 and PTLV-3 [59]. As previously reported, these different nested PCR protocols detect one to five infected cells with a DNA input from 10^5^ cells [59]. We also used this approach to directly detect the potential presence of STLV in samples for which only lymph node tissues were available (n = 15).

For each STLV positive sample for which the *tax-rex* fragment was obtained, attempts to amplify an additional genomic fragment were done to confirm the phylogenetic clustering observed in the *tax-rex* region. A 450 bp LTR fragment for STLV-1 was amplified with a semi-nested PCR using 8255not and LTRU5E primers for the first round and 8255not and 420LTR primers for the second round with the previously described cycling protocol [12,59,60]. Additionally, a 900 bp DNA fragment spanning the *tax-pX*-LTR region was sequenced for each STLV-3 positive sample, as previously reported [12].

#### ***Molecular confirmation of SIV infection***

Attempts to amplify SIV sequences were done for all samples with HIV and/or SIV cross-reactive antibodies as well as for DNA extracted from the 15 samples for which only tissues were available. PCRs were done using consensus-degenerate *pol* primers, Polis4, Polis2 UNIPOL2, and PolOR with the following cycling PCR protocol for the first and second rounds: denaturation at 95°C for 2 min, 20 cycles of denaturation at 95°C for 20 s, hybridization at 45°C for 30 s and extension at 68°C for 1.30 min, 95°C for 2 min and 20 cycles of denaturation at 95°C for 20 s, hybridization at 50°C for 30 s and extension at 68°C for 1.30 min with a final extension at 68°C for 5 min [14].

Following the preliminary phylogenetic analyses, we decided to develop new molecular tools to amplify partial *gag* (470 bp), *env* (500 bp) as well as a longer *pol* fragment (1800 bp) to further analyze SIVrcm genetic diversity. We also developed new primers to amplify a 1900 bp PCR product in the SIVmus *env* gene. All SIVrcm and SIVmus PCR products were obtained by using primers summarized in Additional file 2: Table S1 (see Additional file 2: Table S1).

PCR amplifications were performed using the Long Expand PCR kit (Roche Applied Science, Indianapolis, IN) according to the manufacturer’s instructions. Each amplification reaction included a manual hot-start followed by 35 to 40 cycles. Annealing temperatures were set according to the primer melting temperatures. Extension times varied depending on the size of the expected fragment and were typically set at 1 min/kb.

For all STLV and SIV PCR experiments, reagents were prepared in a dedicated room. PCR products were purified on 1% agarose-gel with a QBIOgene GENECLEAN®Turbo kit (MP Biochemichals). Direct sequencing of both strands using the Big Dye terminator technology (ABI PRISM Big Dye Terminator Cycle Sequencing Ready Reaction kit with AmpliTaq FS DNA polymerase [PE Biosystems, Warrington, England]) was performed on an ABI 3130xl Genetic Analyser. Sequences were then assembled using the software package Lasergene (DNASTAR, Inc., MAD).

### **Phylogenetic analyses**

Newly derived STLV and SIV nucleotide sequences were aligned with reference sequences from the GenBank by using MEGA4 with minor manual adjustments, if necessary [61]. Nucleotide sites that could not be unambiguously aligned were excluded from the analyses. Appropriate models of evolution were selected for each data set using Topali v2.5 software and maximum likelihood phylogenies were reconstructed using PhyML [62,63]. For STLV, analyses were performed using discrete gamma distribution to account for variable substitution rates among sites with four rate categories and the TN93 model. SIV analyses were done under the GTR + Γ_4_ + I model of evolution. Nucleotide frequencies, nucleotide changes rate and gamma distribution shape parameters were estimated from the data. The starting tree was obtained by using PhyML. One thousand bootstrap replications were performed to assess confidence in topology.

### **Sequence similarity plots**

SIVmus partial *env* nucleotide sequence was aligned by using MEGA4 with minor manual adjustments [61]. Sites that could not be unambiguously aligned were excluded. The SIVmus partial *env* sequence was compared to representatives of known HIV/SIV lineages. In order to study whether the newly characterized SIVmus *env* sequence was recombinant with any of the other SIV lineages and more particularly with viruses from the SIVmus/mon/gsn lineage, similarity plot analysis was performed with the SIMPLOT package version 2.5 [64] using a sliding window of 160 nucleic acids (na) moved in steps of 10 na. For this purpose, a bootscan analysis was also realized.

### **GenBank accession numbers**

The new sequences have been deposited in EMBL under the following accession numbers:

STLV-1 partial *tax* sequences (HE589471-HE589483), STLV-1 partial LTR sequences (HE589484-HE589493), STLV-3 partial *tax* sequences (HE591390-HE591393), STLV-3 partial *pX*-LTR sequences (HE591394-HE591396), SIV partial *pol* sequences (HE591397-HE591403), SIVrcm partial *gag* sequences (HE591404-HE591408), SIVrcm partial *env* sequences (HE591409-HE591413), SIVmus partial *env* sequence (HE591414).

## Competing interests

The authors declare that they have no competing interests.

## **Authors’ contributions**

Conceived and designed the experiments: FL, MP, FR, EL. Performed the experiments: FL, VB, FM, CB. Analyzed the data: FL, MP, FR, VB. Contributed reagents/materials/analysis tools: FL, MP, EL, XP, BMO. Wrote the paper: FL, MP, FR, EL. Study conducted in the field: EL, XP, BMO. All authors read and approved the final manuscript.

## Supplementary Material

Additional file 1 Phylogenetic analysis of partial 12S rRNA sequences from Gabonese monkey species. Reference sequences used were as follow: C. cephus (L35191, L35202), C. diana (L35193), C. mona (L35198), C. aethiops (L35187, L35189, L35190), C. nictitans (L35199), C. mitis (L35197), C. patas (L35186), C. neglectus (L35182), C. galeritus (L25208), M. sphinx (L35196), C. torquatus (L35204), Papio species (L35184, L35206), L. atterimus (L35192), M. talapoin (L35205), G. gorilla (L35209), P. paniscus (L35201), P. troglodytes (L35183). The analyses were performed using discrete gamma distribution and TN93 model. The starting tree was obtained by using phyML. One thousand bootstrap replications were performed to assess confidence in topology (only values ≥ 80% are shown and represented by an asterisk). Scale bar represents the number of nucleotide substitution per site. Click here for file

Additional file 2 Primers used to amplify partial sequence of SIVs. Click here for file
