# Supplementary figures and images for: New STLV-3 strains and a divergent SIVmus strain identified in non-human primate bushmeat in Gabon
Source: Retrovirology. 2012 Mar 30;9:28. doi: 10.1186/1742-4690-9-28 (PMC3413610; doi:10.1186/1742-4690-9-28)

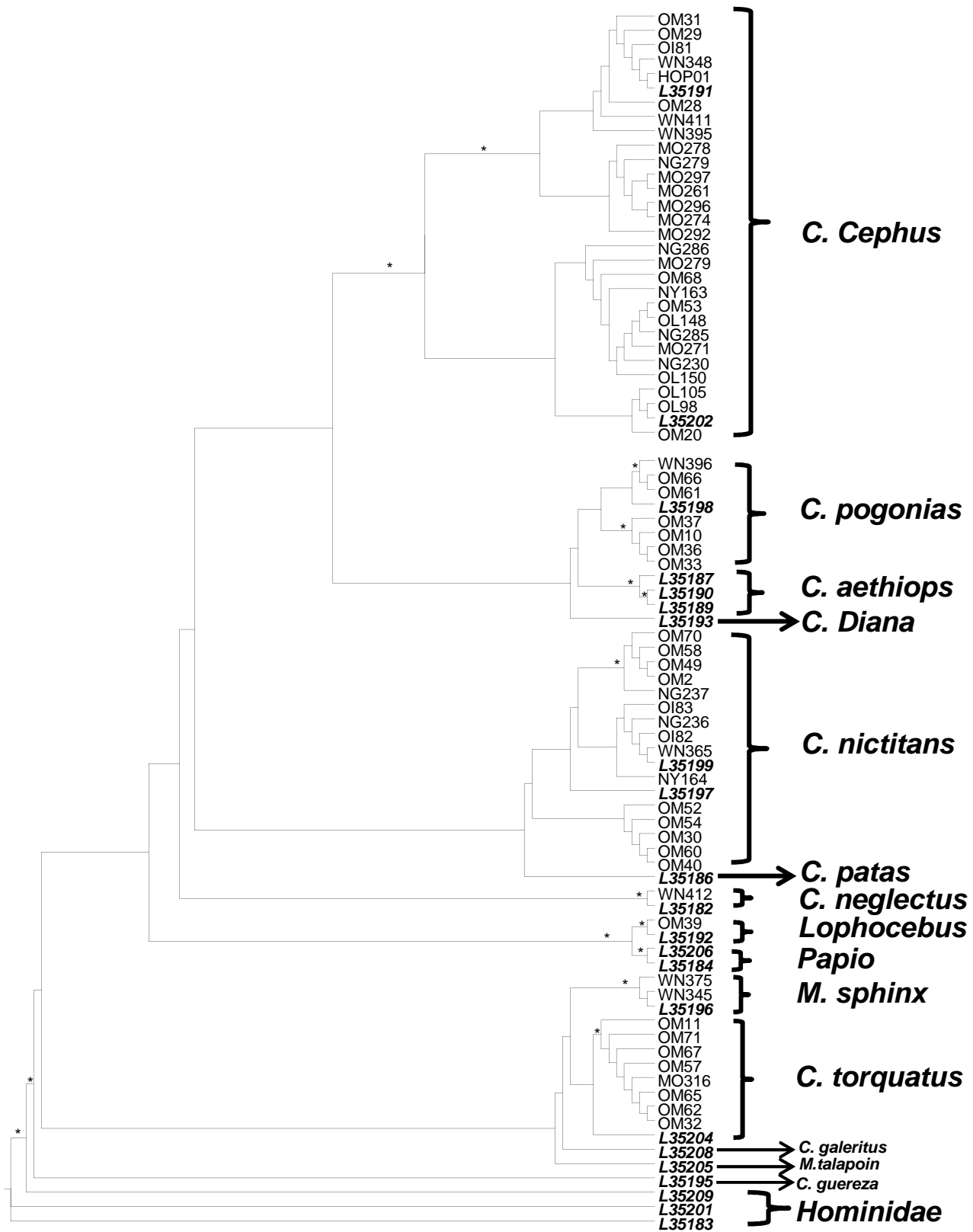

0.05

Supplement: Additional file 1 — Phylogenetic analysis of partial 12S rRNA sequences from Gabonese monkey species. Reference sequences used were as follow: C. cephus (L35191, L35202), C. diana (L35193), C. mona (L35198), C. aethiops (L35187, L35189, L35190), C. nictitans (L35199), C. mitis (L35197), C. patas (L35186), C. neglectus (L35182), C. galeritus (L25208), M. sphinx (L35196), C. torquatus (L35204), Papio species (L35184, L35206), L. atterimus (L35192), M. talapoin (L35205), G. gorilla (L35209), P. paniscus (L35201), P. troglodytes (L35183). The analyses were performed using discrete gamma distribution and TN93 model. The starting tree was obtained by using phyML. One thousand bootstrap replications were performed to assess confidence in topology (only values ≥ 80% are shown and represented by an asterisk). Scale bar represents the number of nucleotide substitution per site. [file 1742-4690-9-28-S1.pdf]
