# Supplementary material for: New STLV-3 strains and a divergent SIVmus strain identified in non-human primate bushmeat in Gabon
Source: Retrovirology. 2012 Mar 30;9:28. doi: 10.1186/1742-4690-9-28 (PMC3413610; doi:10.1186/1742-4690-9-28)
Supplement: Additional file 2 — Primers used to amplify partial sequence of SIVs. [file 1742-4690-9-28-S2.docx]

**Additional File 2**

Table S1: Primers used to amplify partial sequence of SIVs

Samples Primers Tm°C Sequence^a^ (5’-3’) Size (bp)

**SIV partial *pol***

Round 1 PolIS4 54 CCAGCNCACAAAGGNATAGGAGG

PolOR 46 ACBACYGCNCCTTCHCCTTTC

Round 2 PolIS2 56 GGCARATRGAYTGYACNCAYNTRGAA 400

Uni2 59 CCCCTATTCCTCCCCTTCTTTTAAAA

**SIVrcm partial *gag***

Round 1 RCMgagconsF1 50 CTCTTTAATTTGTGCTGCGYACT

RCMgagconsR1 54 TTCCAAAGAGMGAATTGAGGGAG

Round 2 RCMgagconsF2 45 GAAGTGAAAGTGAAAGAYACAGA 470

RCMgagconsR2 47 TGWGTCATCCAATTYTTYACTG

**SIVrcm partial *pol***

Round 1 RCMPolconsF1 50 CTCCCTCAATTCKCTCTTTGG

RCMPolconsR1 55 CTTGGCTYTGAGGATTGTAGGGTA

Round 2 RCMPolconsF2 48 TGGAGAAAGYTAGTAGATTTYAGAGA 1800

RCMPolconsR2 52 CTCTATYTGAGCCCACCAACA

**SIVrcm partial *env***

Round 1 RCMEnvconsF1 55 TACCTTTGGTGCTGGGTTTTCTA

RCMEnvconsR1 55 TCTCTCGAAGCCYTGCCTGAT

Round 2 RCMEnvconsF2 45 ACAGCCCTGACWGTCCA 500

RCMEnvconsR2 53 CTTTGAACTCTTGCCACCCAT

**SIVmus partial *env***

Round 1 OI81_polF1 54 TAGCAAGTCAATGGCCAGTAAGTC

SIVnefas 45 CAGTCCHCCCTTTTCTTT

Round 2 OI81polF4 50 GACCTATCTCCAGGAGAAAGAATA 1900

SIVnefas 45 CAGTCCHCCCTTTTCTTT

^a^ Y= C ou T ; W= A ou T ; R= A ou G ; H= A ou C ou T ; B= C ou G ou T ; M= A ou C S= G ou C ;

K= G ou T ; V= G ou A ou C ; D= G ou A ou T ; N= A ou G ou C ou T
